# Donor and recipient CMV serostatus and antigenemia after renal transplantation: An analysis of 486 patients

**DOI:** 10.1016/j.jcv.2007.10.006

**Published:** 2008-02

**Authors:** David Hughes, John Hafferty, Lee Fulton, Peter Friend, Andrea Devaney, Justin Loke, Ken I. Welsh, Ashok Handa, Paul Klenerman

**Affiliations:** a Nuffield Department of Surgery, John Radcliffe Hospital, Oxford OX3 9TU, United Kingdom; b Nuffield Department of Medicine, Peter Medawar Building, South Parks Road, Oxford OX1 3SY, United Kingdom

**Keywords:** CMV, Transplant, Kidney, Reactivation, Superinfection

## Abstract

**Background:**

Cytomegalovirus infection in renal transplant recipients is a major clinical problem, with both short and long term sequelae. Infection can occur as a result of reactivation of latent virus or new infection from donor tissues. The impact of donor and recipient serostatus on viremia is well recognised, with seronegative recipients at greatest risk after transplantation of an organ from a seropositive donor. However, the impact of grafting such organs into seropositive recipients is less clear.

**Objectives:**

To assess the impact of recipient serostatus on the risk of CMV antigenemia in a large renal transplant cohort.

**Study design:**

We prospectively quantified CMV antigenemia over time in a cohort of 486 recipients. We analysed the antigenemia status according to donor and recipient serostatus.

**Results:**

Antigenemia was most common in seronegative recipients of organs from seropositive donors (D+/R−). Nevertheless, we observed that even in CMV seropositive recipients, the impact of donor serostatus on CMV antigenemia is still substantial (*p* = 0.006; OR = 2.2).

**Conclusions:**

In this large study, donor serostatus clearly plays a significant role in determining CMV risk, even in seropositive recipients.

## 1 Introduction

Cytomegalovirus (CMV) is a significant problem in the immunosuppressed. In particular, renal transplant patients are at high risk of disease in the early post-transplant period. Long term survival of the graft may also be influenced by infection/reactivation (Schnitzler et al., 2003).

CMV may cause disease upon primary infection. Seronegative recipients are therefore at high risk, especially if the donor organ is from a seropositive individual (Brayman et al., 1988; Gjertson, 1992, 2003; Hirata et al., 1996; Ricart et al., 2005; Schnitzler et al., 2003; Warrell et al., 1980). Alternatively CMV may cause disease upon reactivation in immunosuppressed individuals, where immune surveillance is depressed as a result of disease or drug therapy (Rao et al., 2000; Warrell et al., 1980).

To analyse the role of pre-existing immunity in control of CMV post-transplant, we assessed antigenemia in a large cohort of renal transplant patients who had been carefully prospectively followed. The antigenemia assay is a robust quantitative measure of CMV reactivation which has been used in many previous studies (Pancholi et al., 2004). We analysed four patient groups, donor seropositive, recipient seropositive (D+R+), donor seronegative recipient seronegative (D−R−), and the mismatched D+R− and D−R+ groups. Specifically we analysed what the impact of donor serostatus was in recipients who exhibited prior immunity to CMV.

## 2 Methods

The patient cohort was taken from those individuals undergoing renal transplantation at the Churchill Hospital, Oxford. Individuals were routinely tested for CMV serostatus pretransplant using CMV latex assays (BD, Pharmingen). Antigenemia levels were assessed regularly post-transplant up to 99 days, using a cytospin preparation of buffy coat cells from peripheral blood and direct pp65 analysis using APAAP immunochemistry (mouse anti-HCMV pp65 (CLONAB)). The immunosuppressive regimen varied little over this period—all patients received cyclosporin A (dosage adjusted according to levels), methylprednisolone and azathioprine conventional triple therapy. CMV seronegative donors received CMV negative blood products. Prophylaxis for CMV infection (ganciclovir or aciclovir/valaciclovir) was not used during the period studied.

Seven hundred and thirty-five patients were transplanted over the study period. These were divided according to pre transplantation serostatus into four groups—D+R+ (30%), D+R− (23%), D−R+ (24%) and D−R− (23%). Full follow-up data including details of the antigenemia testing up to 99 days were available on 486 patients (Table 1) and it was this group that was analysed in detail.

Statistical analysis was performed using Graphpad Prism software. Analysis of proportion was performed using Fisher's exact tests. To account for multiple comparisons, a *p* value of <0.05/8, i.e. <0.00625 has been used (Bonferroni correction).

## 3 Results

Overall, 35% of patients experienced antigenemia during the 99 day follow-up period, in about half of whom this reached a level of over 5/50,000 cells in blood (Table 1). A smaller fraction reached very high levels of antigenemia, although since this will be influenced by the treatment instituted and the response to therapy, it was not analysed further.

**Fig. 1:**
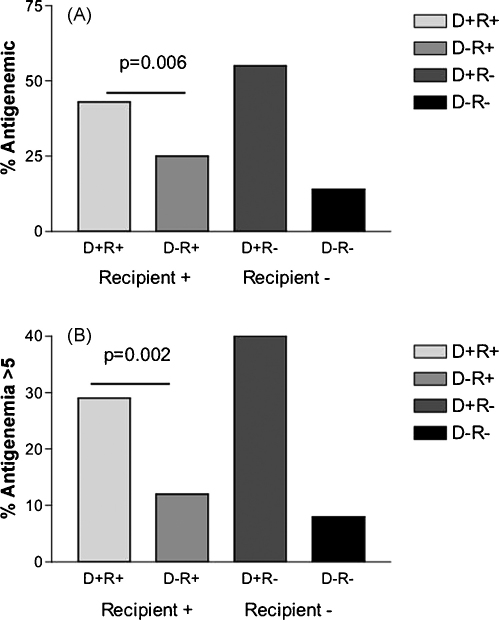
Comparison of CMV antigenemia rates in different clinical risk groups. The upper panel (A) shows the proportion of individuals experiencing CMV antigenemia over the follow-up period in the four different clinical groups. The lower panel (B) shows the frequency of antigenemia at a level >5/50,000 over the same period. The mean onset of antigenemia did not differ between the different groups. The *p* value refers to the impact of donor serostatus in the seropositive recipient group. Other *p* values for these comparisons are shown in Table 2.

Amongst those with antigenemia, the frequencies varied widely between the four patient groups (Table 1 and Fig. 1). The extremes were seen in the seronegative recipient group. Amongst these, those receiving a kidney from a seropositive donor (D+R−) showed an antigenemia rate of 55%, while those receiving an organ from a seronegative donor had a basal rate of 14% (D−R−).

For seropositive recipients, the overall infection rate was 43% in those receiving an organ from a seropositive donor (D+R+), compared to 25% if the donor was seronegative (D−R+). Similarly, for antigenemia levels >5/50,000, the infection rates were 29% and 12%, respectively. The latter represents an odds ratio of 2.9 (*p* = 0.002). While the greatest rate of antigenemia >5/50,000 is seen in the D+R− group, the odds ratio compared to D+R+ is not significant (OR = 1.65; Table 2).

Overall, the risk of infection in R− recipients was 35%, compared to 36% in the R+ group (*p* = n.s.). When analysed by donor serostatus, D+ organs were associated with a 49% infection rate in the recipients, compared to 19% in D− organs (*p* < 0.0001, OR = 4.0). Similarly, when assessing the rate of infection >5/50,000, no significant difference was seen comparing R+ and R− groups (21% vs. 25%, *p* = n.s.), while D+ vs. D− groups showed a major effect (35% vs. 10%; *p* < 0.0001, OR = 4.9). Thus, donor status had a major impact on overall infection outcome, even in a group where about half the recipients were already seropositive.

## 4 Discussion

Much work in the past has identified CMV as a significant complication of renal transplantation, with additional long term consequences in terms of graft survival (Gjertson, 1992, 2003; Hirata et al., 1996; Schnitzler et al., 2003). It is clear that pre-existing immunity modifies the course of infection, as the most significant disease is seen in seronegative recipients who undergo primary infection. It is for this reason that such individuals are often specifically targeted in prophylactic regimens. Nevertheless a significant burden of infection lies outside this group.

The Oxford transplantation programme established at an early stage a regular screening protocol for identifying CMV antigenemia in the recipient cohort, regardless of donor and recipient serostatus. This gave us a valuable data resource with which to tackle the question of how recipient and donor serostatus influence CMV infection/reactivation. Due to the prevalence of CMV in the UK, the proportions of individuals in the four potential groups (D+R+, D+R−, D−R+, D−R−) were roughly equal, thus allowing reasonable comparisons to be made in a large group of patients all undergoing similar well-established regimens of pre- and post-operative monitoring and care (Boeckh et al., 1994; Pancholi et al., 2004; The et al., 1990).

This study suggests that donor serostatus plays a very important role in the outcome of transplantation, and one which may be overlooked in the group of seropositive recipients. Thus even in the R+ group the receipt of a D+ organ increases the risk of CMV antigenemia by two- to threefold compared to receiving a D− organ. This is substantially less than the relative excess risk in the R− group (seven to eightfold), confirming that recipient serostatus also plays a major role, but raises the important issue of to what extent infection seen in this period post-transplant is due to reactivation vs. superinfection.

In principle the D−R− group represents the background infection rate. Sources of error here might include false negatives in D/R serostatus, but if these are truly both negative the infection may represent a nosocomial or indeed a non-hospital source. The infection in the D−R+ group then represents, possibly on top of the background rate, the true reactivation rate. The difference between the D+R+ group and the D−R+ group must represent the superinfection rate. This is substantial, and furthermore the difference between D−R+ and D−R− (i.e. presumed reactivation) is by comparison small and statistically nonsignificant. This suggests that a large burden of disease seen in the R+ cohort is due to superinfection rather than reactivation.

One possible explanation is that pre-existing immunity is relatively strain specific, and that the incoming strain is not efficiently controlled by the combined cellular and humoral responses. There is some evidence for this idea in studies of children born with congenital CMV (Boppana et al., 1999, 2001; Fowler et al., 1992). Here sequence analysis has shown that a superinfecting strain present during pregnancy appears to infect the fetus and cause disease, despite pre-existing antibody (and presumably T cell immunity) in the mother. There is little data on the diversity of strains infecting the transplant population and the cross-protection between them, but this issue will of significance in future studies.

A second interesting possibility is that localisation of virus in the incoming kidney may provide a niche for superinfection. This may be a difficult area for immune responses—since humoral immunity will not provide protection against an infected cell and cellular immunity may be compromised in this organ. The latter may result partly from its anatomy and/or the expression of Class I molecules in epithelial cells. HCMV can exist in replicative form in normal kidneys for extended periods in the face of very strong CD8+ and CD4+ T cells (Griffiths, 1988), as can other pathogens (Ciurea et al., 1999). A further issue in transplant patients may be matching of Class I molecules. HCMV specific CD8+ T cell responses are robust but are focused on specific peptides. If the restricting allele for such a response is not present in the organ then this might further impair the capacity of a host response to contain infection. We investigated the impact of HLA mismatching in this cohort; however, overall we found no influence of the number of matches at Class I or Class II loci on the rate of antigenemia in different D/R groups (data not shown).

Overall these data therefore provide a unique analysis of CMV infection in a large prospectively analysed cohort. On the one hand an important clinical message is that donor serostatus should be considered an important variable in determining outcome even in seropositive recipients, and potentially this group might also benefit in future from CMV prophylaxis in the same way as seronegative recipients—this issue is being addressed in a follow-up study since prophylaxis has been introduced. On the other hand the immunological and virological basis for this enhanced risk of antigenemia requires further analysis, including better data on the diversity of and cross-reactivity between circulating CMV strains. Even with prophylaxis, CMV infection remains an important threat to the transplant recipient and a better understanding of the basis for it might provide new avenues for protection in those at risk.

## Figures and Tables

**Table 1:** Patient characteristics and CMV antigenemia

	D+R+	D−R+	D+R−	D−R−	Total
Patients	128 (26%)	107 (22%)	132 (27%)	119 (25%)	486
CMV detected	55	27	73	17	172 (35%)
CMV >5	29	12	40	8	89 (18%)
CMV >100	7	4	9	3	23 (4%)

**Table 2:** Odds ratios for antigenemia (upper table) or antigenemia >5 (lower table)

Comparison (antigenemia ±)	Odds ratio (OR)	95% confidence interval	*p* value
D+R+ vs. D−R+	2.2	1.28–3.91	0.006
D+R+ vs. D+R−	0.61	0.37–0.99	n.s. (0.049)
D+R+ vs. D−R−	4.52	2.43–8.42	<0.001
D+R− vs. D−R−	7.42	4.00–13.77	<0.001
D+R− vs. D−R+	3.67	2.10–6.39	<0.001
D−R+ vs. D−R−	2.03	1.03–3.97	n.s (0.044)
D+ vs. D−	4.01	2.66–6.04	<0.001
R+ vs. R−	0.81	0.53–1.23	n.s. (0.33)

Comparison (antigenemia >5)	Odds ratio (OR)	95% confidence interval	*p* value
D+R+ vs. D−R+	2.9	1.47–5.89	0.002
D+R+ vs. D+R−	0.61	0.36–1.01	n.s. (0.07)
D+R+ vs. D−R−	4.97	2.28–10.84	<0.001
D+R− vs. D−R−	8.2	3.82–17.60	<0.001
D+R− vs. D−R+	4.85	2.47–9.54	<0.001
D−R+ vs. D−R−	1.69	0.69–4.13	n.s. (0.2)
D+ vs. D−	4.9	2.95–8.17	<0.001
R+ vs. R−	0.99	0.66–1.49	n.s. (1.0)

Data are derived from Fig. 1 and Table 1. Fisher's exact test was used to calculate statistical significance. To account for multiple comparisons, a *p* value of <0.05/8, i.e. <0.00625 has been used (Bonferroni correction).
